# Mapping potential wildlife habitats around Haro Abba Diko controlled hunting area, Western Ethiopia

**DOI:** 10.1002/ece3.7913

**Published:** 2021-07-14

**Authors:** Mosissa Geleta Erena, Tekalegn G. Yesus

**Affiliations:** ^1^ Department of Biology Wollega University Nekemte Ethiopia; ^2^ Department of Remote Sensing and Geo‐Informatics Wollega University Nekemte Ethiopia

**Keywords:** African buffalo, controlled hunting, Ethiopia, GIS, Haro Abba Diko, wildlife habitats

## Abstract

Remote sensing and geographic information system technologies provide useful data to analyze and map potential wildlife habitats based on physical parameters collected from the field. HADCHA was established with a total area of 20,000 ha, while many more comparable potential wildlife habitats were left outside the area. This study aims to identify and map potential wildlife habitats around HADCHA. Data were collected using Landsat 5 thematic mapper and Sentinel‐2A satellite image, a digital elevation model with 30 m pixels downloaded from ASTER data, and existing GIS Shapefile layers. Thematic Mapper data were downloaded from USGS and processed with Erdas Imagine 2015 software. To evaluate potential wildlife habitat around HADCHA, habitat suitability parameters such as settlement, slope, water, and road buffer zones were used for habitat evaluation and mapping. Accordingly, 16,795 ha of potential wildlife habitats were identified and mapped on westwards of HADCHA. In the new PPWH, about 476.68 ha (2.84%) were moderately suitable, 14,119.17 ha (84.04%) suitable and 2,200.08 ha (13.10%) highly suitable but only 4.2 ha (0.02%) identified as unsuitable. Legal protection of the PPWH around HADCHA could increase the conservation of African buffalo, other mammals, and their habitats. While the mapped potential wildlife habitats had the potential to be parts of HADCHA, it was neglected and has not yet obtained conservation attention. The finding appeals for legal protection of the PPWH and expansion of HADCHA, which could maximize the conservation efforts taken to wild animals of the area. Neglecting this potential wildlife habitat for a long period of time exposed African buffalo and other large‐sized mammals to illegal hunting practices. Policymakers and conservationists shall revise and design the future action plan of HADCHA on how to expand the current‐controlled hunting area and maximize revenue generation from African buffalo and other potential trophy species of the area.

## Graphical abstract

Potential wildlife habitat used for trophy hunting.

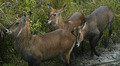

## 1 INTRODUCTION

Protected areas (PAs) are planned and managed with the prime objective of biodiversity conservation across the globe (Bruner et al., 2001). Moreover, PAs provide different direct and indirect ecosystem services (Schreckenberg et al., 2016). Because of its diverse significance, the number of PAs has been increasing and almost doubled in the past two decades (Deguignet et al., 2014). To enhance the future conservation of biodiversity, mapping neglected but potential wildlife habitats have a vital role for sustainable conservation.

Potential wildlife habitat is a habitat that supports the needs of wildlife in or around protected areas. Geographical Information System (GIS) is used to study potential wildlife habitats around protected areas, vegetation cover, land use dynamics, and biodiversity of protected areas (Ohmann et al., 2011). With advanced technology in mapping tools, vegetation maps are effectively developed and successfully modernized using many GIS methodologies and remote sensing data (Bargiel & Herrmann, 2011; Ohmann et al., 2011). GIS has a powerful tool to collect, store, maintain, transform, and display spatial data of a species (Markon, 1994). It can address complicated problems, analyze wide ranges of data and other issues in a more comprehensive way (Danks & Klein, 2002). An increase in the availability of remotely sensed data has a vital role in the application of habitat mapping (Rushton et al., 2004). GIS and remote sensing have been used to determine the habitats of mammals and birds (Danks & Klein, 2002).

Remote sensing can be used to identify potential wildlife habitats for the purpose of conservation and management activities of wildlife. However, it requires detailed knowledge of the locality, as well as throughout the country (Rushton et al., 2004). Knowledge of species of interest can be used to design strategies for managing species habitat and their population dynamics (Rushton et al., 2004). Knowledge of the target species is also important when the habitat of a species encompasses the habitat of other plant and animal species which could be important to design conservation and management efforts for an entire ecosystem.

**FIGURE 1:**
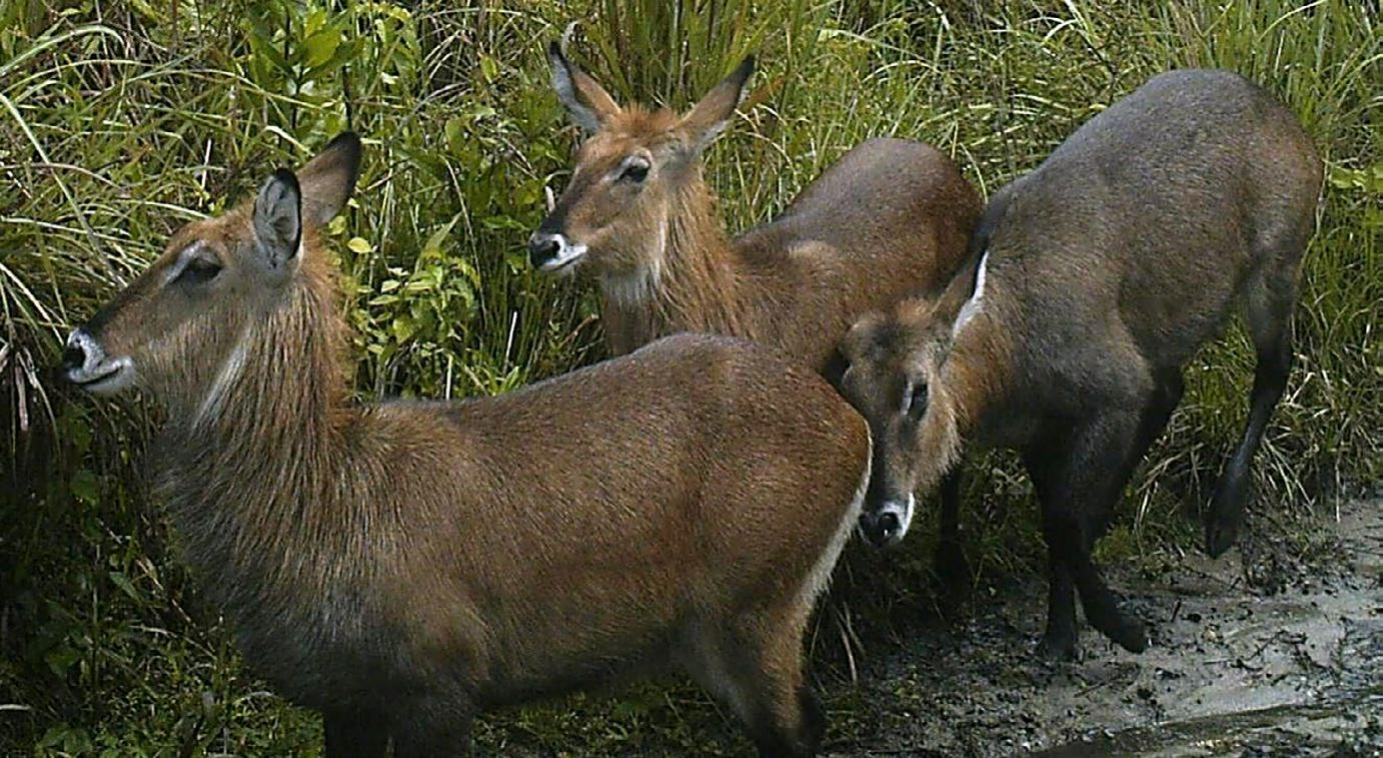
A Herd of waterbuck in the proposed potential wildlife habitat around HADCHA

African buffalo (*Syncerus caffer*) is considered as umbrella species in Haro Abba Diko Controlled Hunting Area (HADCHA) because conserving this species and its habitat indirectly protects many other species that make up the ecological community of the area. It has got special attention because of its trophy value in the area and in Ethiopia. African buffalo has been widely distributed in the Didessa River Valley and HADCHA. HADCHA was established in 2010 by excluding more potential wildlife habitats in the adjacent areas. However, these potential wildlife habitats have not been yet documented for the future expansions of HADCHA. Moreover, there is no well‐organized land use plan analyzed for current and future conservation and management strategies of African buffalo and other medium and large‐sized mammals of the area. Some of the common large mammals that exist in the area are waterbuck (*Kobus ellipsiprymnus*), lion (*Panthera leo*), common warthog (*Phacochoerus africanus*), bushbuck (*Traglaphus scriptus*), bushpig (*Potamochoerus larvatus*), leopard (*Panthera pardus*), hyena (*Crocuta Crocuta*), Giant forest hog (*Hylochoerus meinertzhageni*), and Olive baboon (*Papio Anubis*). Among these, antelopes such as waterbuck are commonly hunted for bushmeat by the local community (Figure 1). Currently, African buffalo is threatened by illegal trophy hunting and poaching for meat in and around HADCHA. In addition, African buffalo habitats are severely threatened by formal settlement, agricultural expansion, and encroachment (Erena et al., 2019). Hence, efforts made to map more potential habitats around HADCHA and conservation of African buffalo habitats could promote the conservation of the entire wildlife in the area.

The main hypotheses in this study area are the potential areas around HADCHA have similar biological and physical characteristics that can fulfill habitat requirements of African buffalo and other large mammals. Around HADCHA, there are wider potential wildlife habitats that could be used for the purpose of biodiversity conservation. Hence, potential wildlife habitats need to be mapped for the expansion of HADCHA. This study, therefore, aims to map the potential wildlife habitats around HADCHA which could be helpful for policymakers to develop action plan for sustainable wildlife conservation and land use planning of the area. Mapping more potential wildlife habitats around HADCHA could enhance conservation of the umbrella species, the African buffalo, and other trophy species of the area as well as the entire wildlife habitats.

## 2 MATERIALS AND METHODS

### 2.1 Description of the study area

**FIGURE 2:**
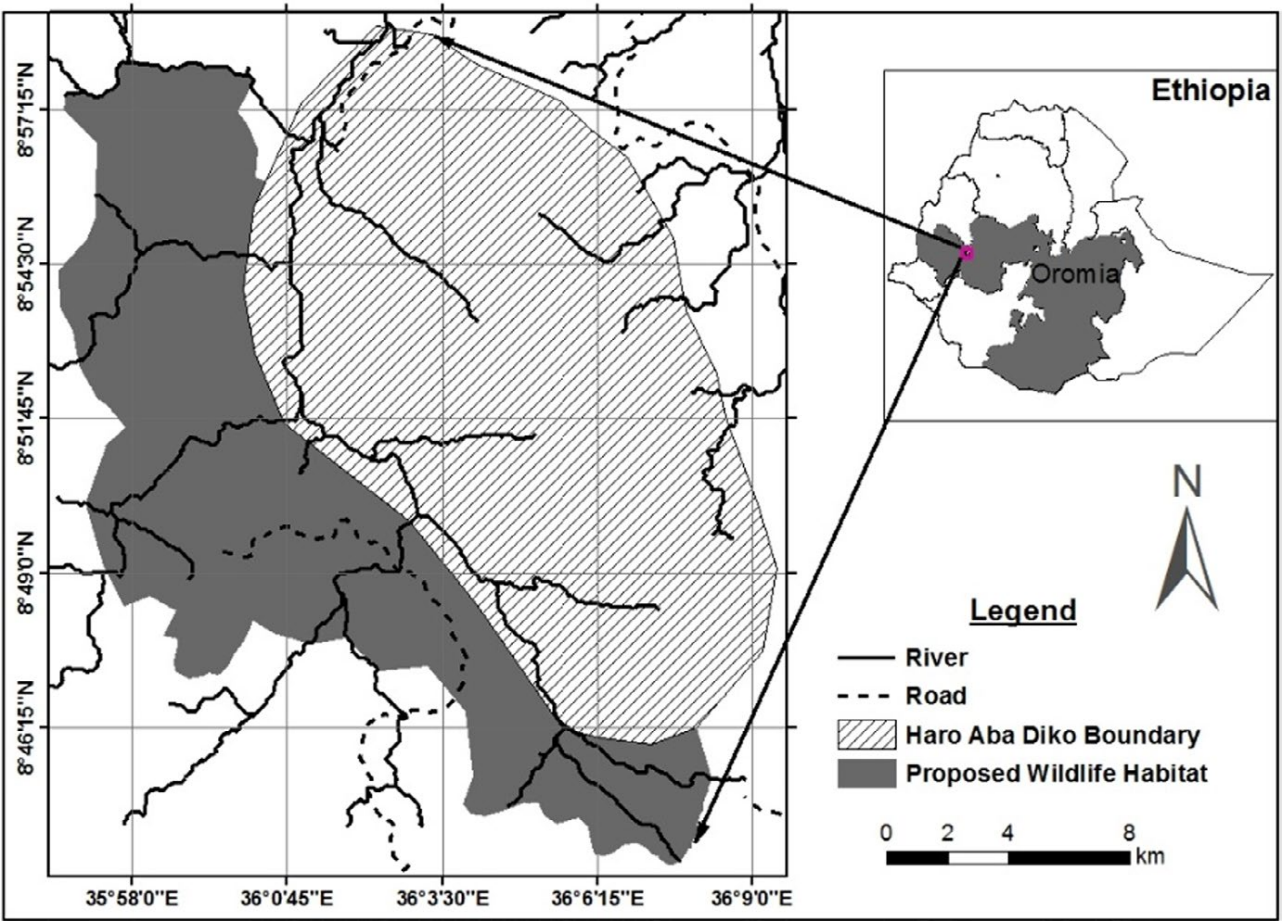
Location map of the study area and the PPWH adjacent to HADCHA

HADCHA is located in the Buno Bedele Administrative Zone of Oromia Regional State, Ethiopia. It is approximately 418 km southwest of Addis Ababa, along the Addis Ababa–Nekemte–Bedele or Addis Abbaba–Jimma–Bedele road. It is located in the Dabo Hana district of Buno Bedele Administrative Zone. HADCHA is situated between 8°40′20″ to 8°48′06″N latitude and 35°48′01″ to 35°56′40″E longitude with an elevation ranging from 1,780 to 2,584 m asl (Figure 2). HADCHA is part of the former Didessa Wildlife Sanctuary and other potential areas in the Didessa‐Dabena Valleys. It was established for the purpose of practicing only trophy hunting of African buffalo and conservation of the area, while many more trophy species exist in the area. Legal trophy hunting of African buffalo was limited to HADCHA, though the spatial distribution of this species exceeds beyond the area demarcated as a controlled hunting area. The potential ranges of African buffalo were found on the western side of HADCHA such as Qoddi Gassi and along the Dabena river valley (Erena et al., 2019). The study area is characterized by lowland climatic condition and receives a unimodal annual rainfall. The wet season extends from April to October with the highest rainfall recorded from June to September. The other months were categorized as dry season though they received little rainfall. The mean annual rainfall of the area from 2007 to 2017 was 1,434.1 mm. The mean monthly maximum temperature was 35.2℃ recorded in May, and the mean minimum was 12.3℃ in January.

### 2.2 Methods

#### 2.2.1 Sighting locations of African buffalo

The study was carried out from May 2019 to June 2020 in Haro Abba Diko Controlled Hunting Area and the surrounding potential wildlife habitats covering both the wet (April–September) and dry (October–March) seasons. An on‐foot field survey was carried out in HADCHA and accessible potential wildlife habitats assumed to host African buffalo and other large mammals around HADCHA. During the survey, evidences for the presences of African buffalo in the surveyed habitat were recorded using direct and indirect evidences. African buffalo detection sites were used as a leading criterion to map and propose potential wildlife habitats around HADCHA. African buffalo is considered as an “umbrella” species of the area because the conservation and management efforts of this species encompass the habitat of numerous plants, animals, and the entire ecosystem. The distribution of African buffalo around HADCHA was confirmed by the ground survey method. During the survey, all potential buffalo areas around HADCHA were identified by the guidance of local residents. The other buffalo detection sites were identified during the ecological studies of African buffalo which was conducted in 2017/18. Accordingly, sightings of African buffalo were recorded through indirect evidence such as dung and footmarks, and marked using GPS. Besides, savanna woodland and forest habitats that could be used by African buffalo and other medium and large‐sized mammals were identified as potential wildlife habitats around HADCHA. To delineate the Proposed Potential Wildlife Habitat (PPWH) around HADCHA, supervised (129 ground truth data), and on‐screen image interpretation (satellite image and Google Earth) methods used. The best habitat characteristics of HADCHA were used as benchmark criteria to evaluate the habitat suitability of the PPWH as described by Sarell and Haney (2003). Benchmark habitat features are the best habitat characteristics in a given habitat against which all other habitats are rated and evaluated. Habitat suitability is the ability of the habitat in its current condition to support a species (Sarell & Haney, 2003). Hence, potential wildlife habitats were marked using the best habitat features that affect the survival of wild mammals such as vegetation cover, slope, and the proximity of perennial water, buffer zone to settlements, and buffer zone to the road (Kushwaha et al., 2001).

#### 2.2.2 Remote sensing and GIS data analysis

**FIGURE 3:**
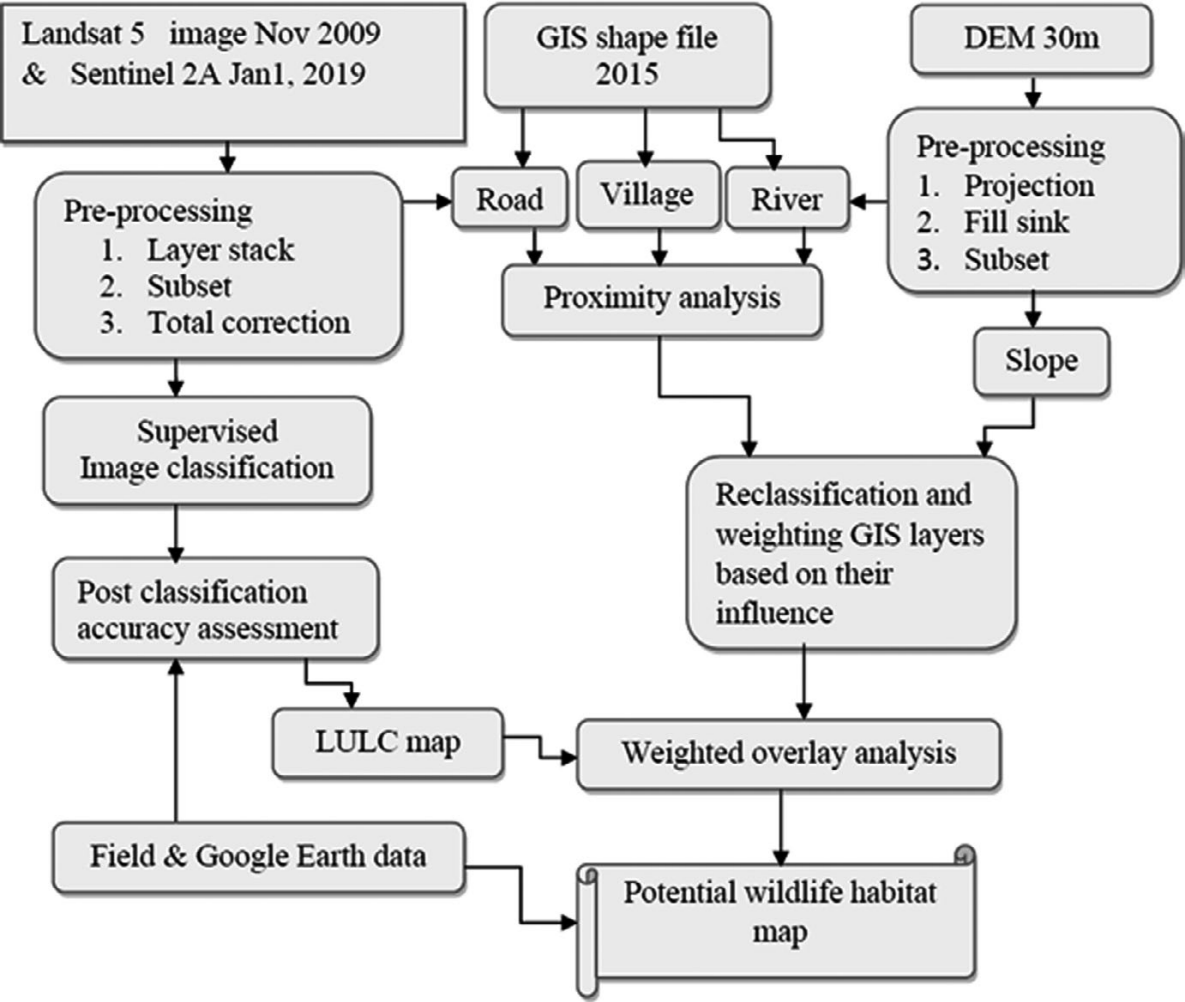
Schematic flow chart of mapping potential wildlife habitat around Haro Abba Diko Controlled Hunting Area

To map the potential wildlife habitats of the area, landsat 5 thematic mapper, Sentinel‐2A satellite image, a digital elevation model with 30m pixels downloaded from ASTER data, and GIS shapefile layers were used. The landsat data were acquired from the USGS official data product website (https://earthexplorer.usgs.gov/) scanned by TM sensor on November 18, 2009. The specific product type of this sensor data was acquired on Nov 18, 2009 (Table 1). The general flow chart of the overall method is described in Figure 3.

**TABLE 1:** Spectral band of Sentinel‐2A satellite image used with its resolution

Sentinel‐2 bands	Wave length (µm)	Resolution (m)	Band width (nm)
Band 1‐coastal aerosol	0.443	60	20
Band 2‐Blue	0.490	10	65
Band 3‐Green	0.560	10	35
Band 4‐Red	0.665	10	30
Band 5‐Vegetation Red Edge	0.705	20	15
Band 6‐Vegetation Red Edge	0.740	20	15
Band 7‐Vegetation Red Edge	0.783	20	20
Band 8‐NIR	0.842	10	115
Band 8A‐Narrow NIR	0.865	20	20
Band 9‐Water vapor	0.945	60	20
Band 10‐SWIR circus	1.375	60	20
Band 11‐SWIR	1.610	20	90
Band 12‐SWIR	2.19	20	180

An optical remote sensing Sentinel‐2A can support a wide range of land use studies and geophysical applications. Sentinel‐2A saves the time required to collect data, build a rich, and relatively clear data (Drusch et al., 2012; Li & Roy, 2017; Whitcraft et al., 2015). A Sentinel‐2 image is a compilation of granules of fixed length (approximately 25 km across the track and 23 km along the track) and an area of 100 km^2^ in UTM/WGS84 projection (Drusch et al., 2012; Li & Roy, 2017). For this study, the selected bands of Sentinel‐2A used for stacking were band 2, 3, 4, 8, 11, and 12 which are corresponding to Landsat bands of blue, green, red, near‐infrared, shortwave infrared 1, and shortwave infrared 2, respectively (ESA, 2015). This optical remote sensing group Sentinel‐2A multispectral instrument has high spatial resolution and is easily used to identify natural habitats from human‐modified habitats (Li & Roy, 2017).

#### 2.2.3 Preprocessing of Sentinel‐2A multispectral instrument

Sentinel‐2 is an Earth observation mission developed by European Space Agency (ESA) and built by Airbus Defense and Space as part of the Copernicus Program to perform terrestrial observations as forest monitoring, land cover changes detection, and natural disaster management (Sentinel‐2, MSI, 2015). It consists of two identical satellites, Sentinel‐2A and Sentinel‐2B (Lanaras et al., 2018). Sentinel‐2 comprises 13 spectral channels with a 290 km swath and spatial resolutions of 10 m (four visible and near‐infrared bands), 20 m (six red‐edge/shortwave infrared bands), and 60 m (three atmospheric correction bands) as shown in Table 1 (Nigussie et al., 2019). Cloud‐free Sentinel‐2A satellite image was downloaded on January 2019 from the official website of Sentinel data hub (http://www.copernicus.eu) and assigned a common output projection (UTM/WGS84) and resolution using the IMPACT‐Toolbox software (Simonetti et al., 2015). Level‐1C processing includes radiometric and geometric corrections include ortho‐rectification and spatial registration on a global reference system with subpixel accuracy. Lower spatial resolution bands were resampled using bilinear resampling method to 10m for stacking (Nigussie et al., 2019). The Top of Atmospheric Reflectance Byte (TOARB), that is (0, 255), value was calculated as the ratio of the resampled image radiometric resolution from 12‐bit to unsigned 16‐bit integer data and then to TOARB using linear transformation equation, which was mathematically expressed as TOARB = (DN × 0.0255) as described by Sentinel‐2, MSI (2015), where:

TOARB is Top of Atmospheric Reflectance Byte
DN is Digital Number of the original image (0–4095)
0.0255 is the image relative reflectance conversion factor 10^(−4)^

The preprocessed multispectral instrument Sentinel‐2A data were subset in Erdas Imagine 2015 software by using study area GIS shapefile for land use land cover mapping and feature extraction.

#### 2.2.4 Image acquisition and analysis of land cover types

Thematic Mapper data with path 170/54 row/path were downloaded from USGS and processed by Erdas Imagine 2015 software. The data were preprocessed in both radiometric and geometric methods to improve the quality of the image. Bands from 1 to 5 and 7 were layer stacked, and the image subset was done using the study area. The land use/land cover (LULC) of the study area was generated from Landsat 5 and Sentinel‐2A multispectral satellite images. Supervised based maximum likelihood image classification algorithm was used in false color combination (layer 4, layer 3, and layer 2) of satellite image to extract LULC map of the study area. These layers are the most common image combinations in land use/land cover mapping and change detection for optical remote sensing. LULC was analyzed using Erdas imagine 2015 software for Haro Abba Diko controlled hunting area and the PPWH. Images of the study area were obtained during the dry season to get a clear image of land cover types. In the present study, four major land use/ land cover types such as forest, savanna woodland, and farmland were identified. These land use/land cover types were identified with close association of pixels on satellite images and data obtained from 129 ground truth collected from different study sites. Ground truth points were used as training sites to identify each land use/land cover type which is used to ensure the accuracy of supervised classification and interpretation of the results. The other land cover types (shrubland, bushland, glades, and bamboo) were represented as other vegetation covers (Table 2).

**TABLE 2:** Descriptions of land cover types in the study area

Land cover types	Descriptions
Savanna woodland	It mainly contains *Combretum* and *Commiphora* species mixed with *Acacia* species. Grass species such as *Hyparrhenia* species and *Setaria poiretiana* were dominant in savanna woodland habitats
Forest	It is covered by natural forests such as riverine and riparian vegetation
Farmland	It includes natural habitats modified by humans such as croplands, human settlements, grazing land, and fallow lands of not more than one year
Other vegetation covers (shrubland, bushland, glades, and bamboo)	It includes bushlands, shrublands, and other grassland habitats mixed with bamboo and a few scattered trees

The accuracy assessment of 2009 land use land cover classification was done using historical Google Earth image data. LULC classification accuracy result was done through data collected by GPS and Google Earth with high‐resolution images. Sample points were collected from both field and Google Earth pro data, and it was converted into shapefile and overlay into the classified images. Finally, the reference data were coded with classified data and accuracy result was assessed automatically in Erdas Imagine 2015 software including kappa coefficients. The overall accuracies for the years 2009 and 2019 were 85.19% and 91.4%, respectively.

### 2.3 Habitats features used in potential wildlife habitat mapping

#### 2.3.1 Suitability of slope

Considering large and medium‐sized mammals as species of concern, slope suitability classes were classified at different intervals with 0–5° steepness as highly suitable and slope >35° as highly unsuitable. Slope with the lowest degree is assumed to be more suitable for large mammals than slope with the highest degree (Goparaju et al., 2017; Kushwaha & Roy, 2002). The slope is the maximum rate of change from a cell to its neighbors, which is mainly used to measure the steepness of the terrain. It is calculated as the ratio of the vertical change to the horizontal change for any two distinct points on a line (Barringer & Lilburn, 1997). Slope measures the rate of change of elevation at a surface location and expressed in degree (°). The slope of the study area was generated from ASTER data called Digital Elevation Model (DEM) with a 30 m spatial resolution. Unprocessed DEMs have artifacts such as depressions and peaks resulted from DEM acquisition process, which needs to be removed before further analysis (Arefi & Reinartz, 2011; Santillan & Santillan, 2014). These data were projected to the common coordinate system of Ethiopia (WGS 1984 UTM zone 37), and the peaks and sinks are also filled by GIS processing tools. The slope for this study area was classified into five classes as described by Goparaju et al. (2017). These include gentle slope (0–5), moderate (5–10), strong (10–25), steep (25–35), and very steep (>35). Slope with the highest degree has given less weight whereas those with the smallest degree have given more weight meaning they are more suitable for wild animals.

#### 2.3.2 River buffer estimation

The perennial river buffer zone for both HADCHA and the PPWH was determined at the 0.5km scale interval assuming that mammal preference for habitat decreases as distance increases from river or water sources. The drainage layer was extracted from DEM of the study area using Arc GIS hydrology too. This is used to map the distance that wild animals need to travel to get water and identify habitats more suitable for animals. The river buffer zones were measured using Euclidean distance using Arc GIS software spatial analyst. The perennial river buffer zone was scaled as 0–0.5, 0.5–1, 1–1.5, 1.5–2.5, and >2.5 km and ranked as highly suitable, suitable, moderately suitable, slightly suitable, and unsuitable, respectively.

#### 2.3.3 Settlement buffer zone

The location of each village around the study area was identified by using GIS tools, field surveys, satellite images, and toposheet of the study area. The settlement buffer zone was identified using Euclidean distance using Arc GIS software spatial analyst. According to Goparaju et al. (2017), wildlife habitat should be far from agriculture, settlements, towns, and fallow lands. Hence, the most suitable land use types exist far from settlement areas. Different weight values were assigned to habitats to determine their level of suitability for wildlife.

#### 2.3.4 Road buffer zone

Roads were extracted from Sentinel‐2A satellite image with the overlay of shapefile as per the study area using Arc GIS software. The proximity of a buffer zone to roads was generated using Euclidean distance using Arc GIS spatial analyst tools. Buffer to roads was roughly classified into four classes based on the assumed impact they have on wildlife habitats (Table 3).

**TABLE 3:** Different weight values assigned to the core wildlife habitat buffer classes in the study area

Key factors affecting wildlife habitat suitability	Description	Wildlife habitats with assigned suitability ranks in parenthesis				
	Rank	1	2	3	4	5
	Classification	Highly suitable	Suitable	Moderately suitable	Slightly suitable	Unsuitable
Slope	Degree	0–5	5–10	10–25	25–35	>35
River	km	0–0.5	0.5–1	1–1.5	1.5–2.5	>2.5
Road	km	>7	5–7	2.5–5	1–2.5	0–1
Settlement	km	>10	7–10	4–7	1.5–4	0–1.5

#### 2.3.5 GIS‐based suitable wildlife habitat analysis

A multi‐criteria decision making/analysis (MCDM/A) called weighted overlay (WO) is used to overlay several rasters using a common measurement scale and weights based on its importance (Chaudhari et al., 2018; Zeinolabedini & Esmaeily, 2015). The WO tool is the most used approach for overlay analysis to solve multi‐criteria problems such as site selection and suitability models (Aksoy & San, 2019). Proximity to settlement/village, roads, and river were analyzed in Arc GIS spatial analysis tools to identify potential wildlife habitats. Land use land cover and slope values were also considered to evaluate the suitable site for wildlife habitats. Each layer was classified and reclassified based on their influencing factors to identify wildlife habitats (Ahmad et al., 2018; Goparaju et al., 2017).

## 3 RESULTS

### 3.1 Sighting locations of African buffalo

**FIGURE 4:**
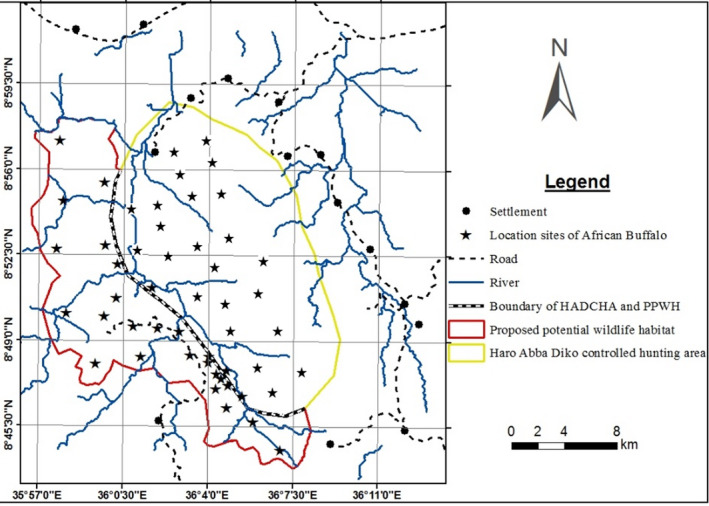
Sighting locations of African buffalo in HADCHA and the surrounding PPWH

African buffalo are mostly recorded along the Dabena river valley during the dry season and spread more during the wet season (Figure 4). During the wet season, African buffalo were recorded at longer distances from HADCHA and Dabena river valley such as Qoddi Gassi of Meko district and westwards of Chewaka district.

### 3.2 Habitats features used in wildlife habitat mapping

As the study area has ample perennial rivers, only few habitats are recognized to have a minimum required distance, 2.5 km, away from the nearby rivers. Moreover, the northern and eastern periphery of HADCHA was surrounded by indigenous community and new settlers that came from the eastern parts of Ethiopia as a drought‐driven resettlement program. Thus, periphery of the area was used for subsistence agriculture and livestock grazing. However, the newly PPWH was free from settlement and buffer zone was demarcated at Euclidian distance of 5 km from the surrounding human residences and farming lands.

### 3.3 The surrounding habitat features of HADCHA

**FIGURE 5:**
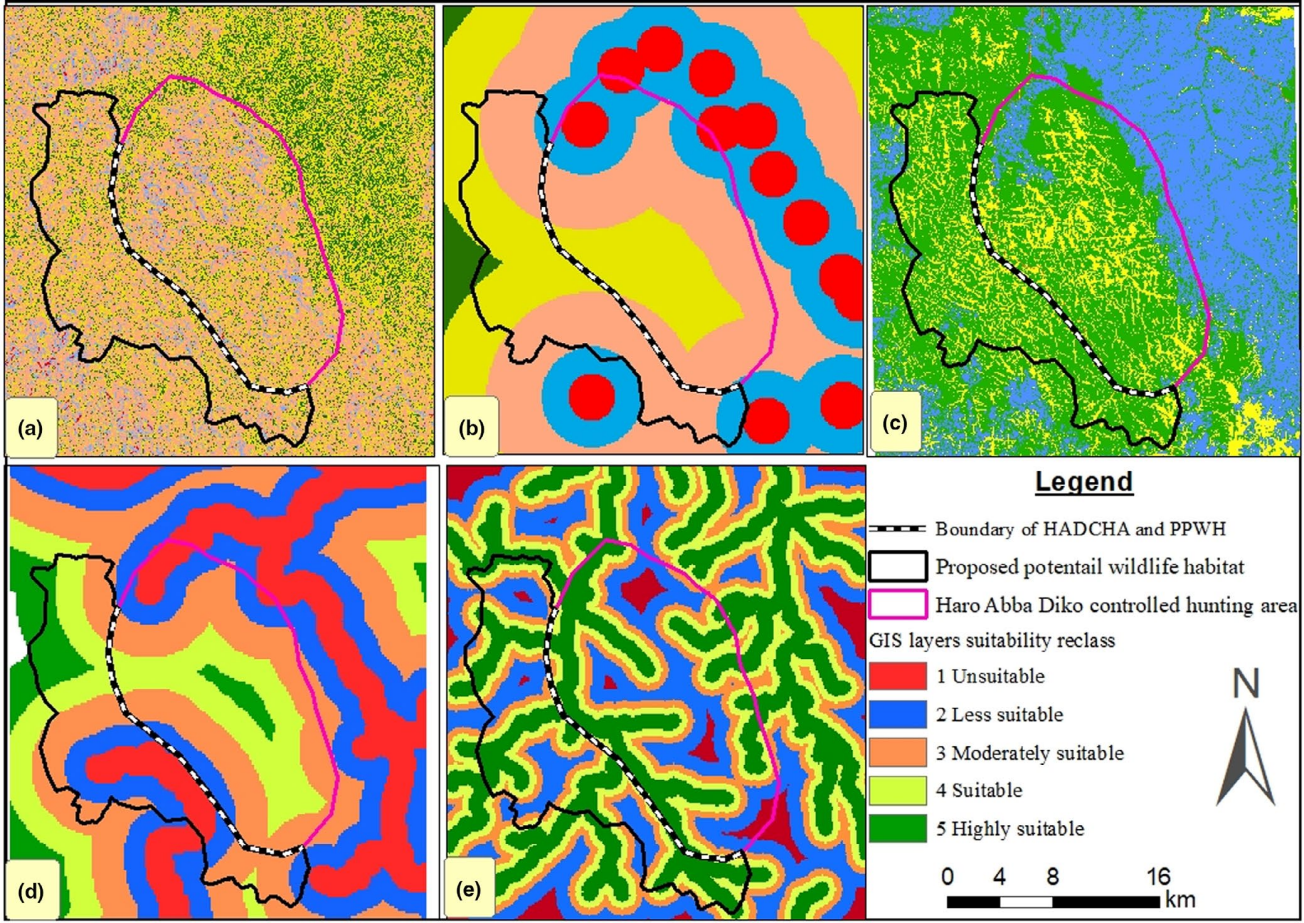
GIS layer reclassification for habitat suitability analysis: (a) Slope, (b) Settlement, (c) Land use land cover, (d) Road, and (e) Perennial River

**FIGURE 6:**
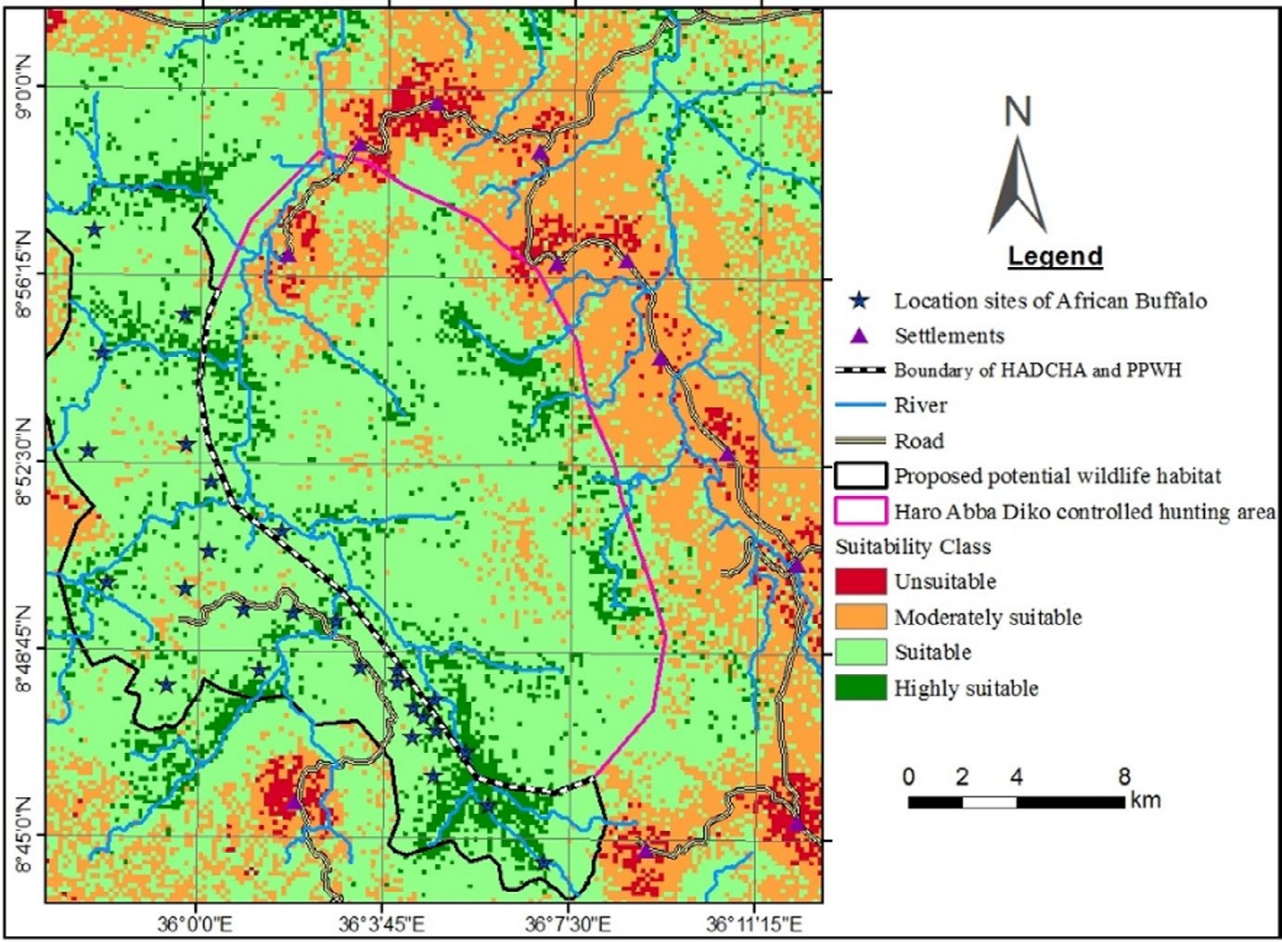
Potential wildlife habitat suitability map of HADCHA and PPWH in 2019

Wildlife habitat suitability map of HADCHA and PPWH is indicated in Figure 5. In HADCHA, 2,309.40 ha (9.14%) was unsuitable, 1,049.76 ha (4.15%) moderately suitable, 19,445.86 ha (76.99%) suitable and the remaining 2,463.68 ha (9.75%) highly suitable. However, in the newly PPWH, only 4.20 ha (0.02%) was unsuitable, but 476.68 ha (2.84%) was recognized as moderately suitable, 14,119.17 ha (84.04%) suitable, and 2,200.08 ha (13.10%) highly suitable (Table 4; Figure 6).

**TABLE 4:** Evaluation of wildlife habitat suitability classes in HADCHA and PPWH in 2019

Suitability classes	HADCHA		PPWH		Total(ha)
	ha	%	Ha	%	
Unsuitable	2,309.40	9.14	4.20	0.02	239.83
Moderately suitable	1,049.76	4.15	476.68	2.84	3,600.58
Suitable	19,445.90	76.99	14,119.17	84.04	33,565.60
Highly suitable	2,463.68	9.75	2,200.08	13.10	4,663.76
Total	25,268.70	100.03	16,800.13	100.00	42,069.8

### 3.4 Land use/land cover types analysis

**FIGURE 7:**
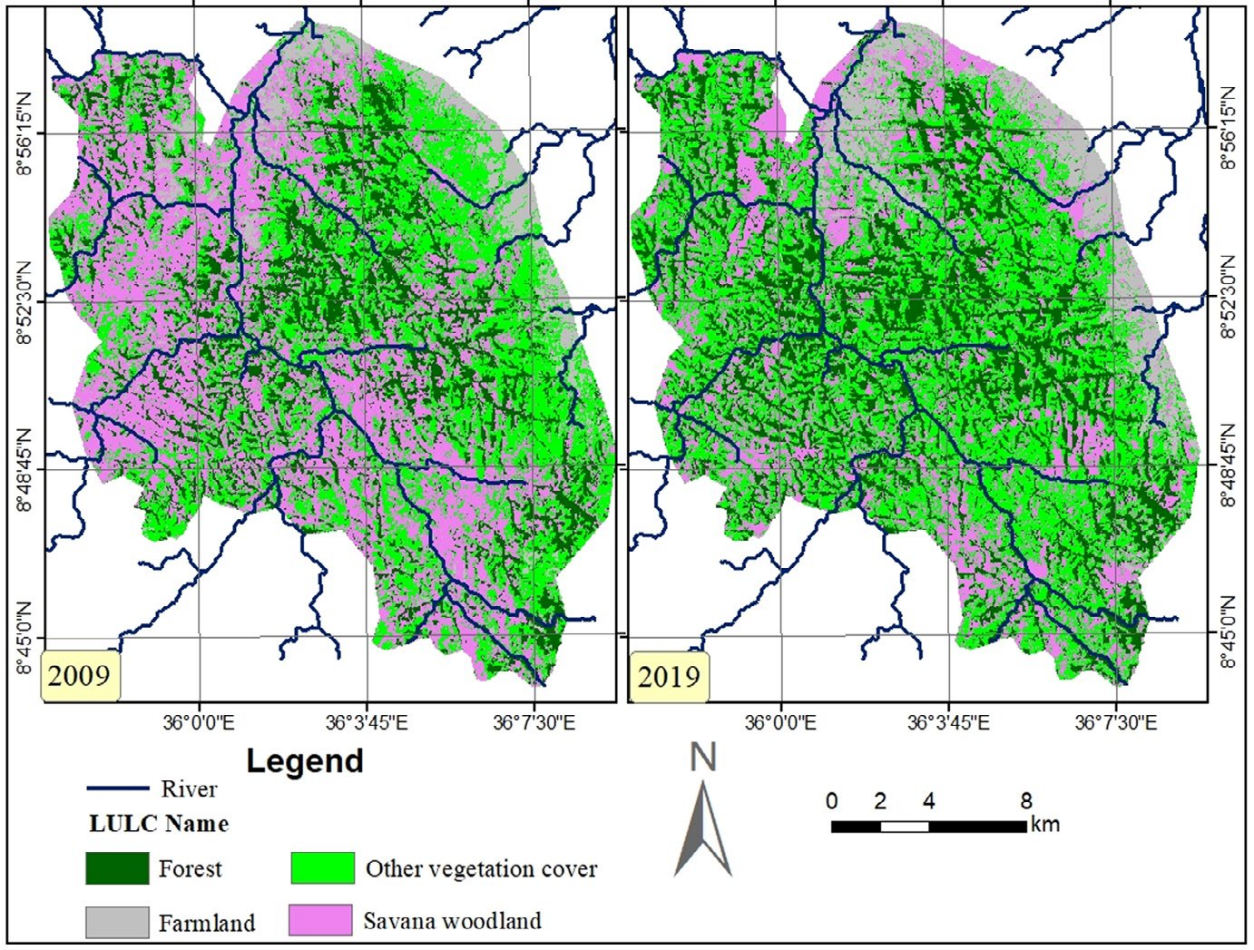
Land use land cover types of HADCHA in the years 2009 and 2019

In HADCHA, savanna woodland was the dominant land cover type, followed by other vegetation types (shrubland, bushland, glades, and bamboo) and forest. Savanna woodland decreased by 2,934.61 ha (35.14%) between the years 2009 and 2019 (Table 5). However, cultivated and forest areas increased by 885.39 ha (38.34%) and 22.70 ha (1,235.57%), respectively, between the years 2009 and 2019. Also, other vegetation types increased by 813.68 ha (8.88%) during the same periods (Figure 7).

**TABLE 5:** Land use land covers types of HADCHA as determined from satellite images of 2009 to 2019

Land cover types	2009		2019		2009–2019	
	ha	%	ha	%	ha	%
Savanna woodland	8,350.70	33.05	5,416.09	21.43	−2,934.61	−35.14
Forest	5,442.40	21.54	6,677.97	26.43	1,235.57	22.70
Farmland	2,309.40	9.14	3,194.76	12.64	885.39	38.34
Other vegetation covers	9,166.20	36.27	9,979.88	39.50	813.68	8.88

**FIGURE 8:**
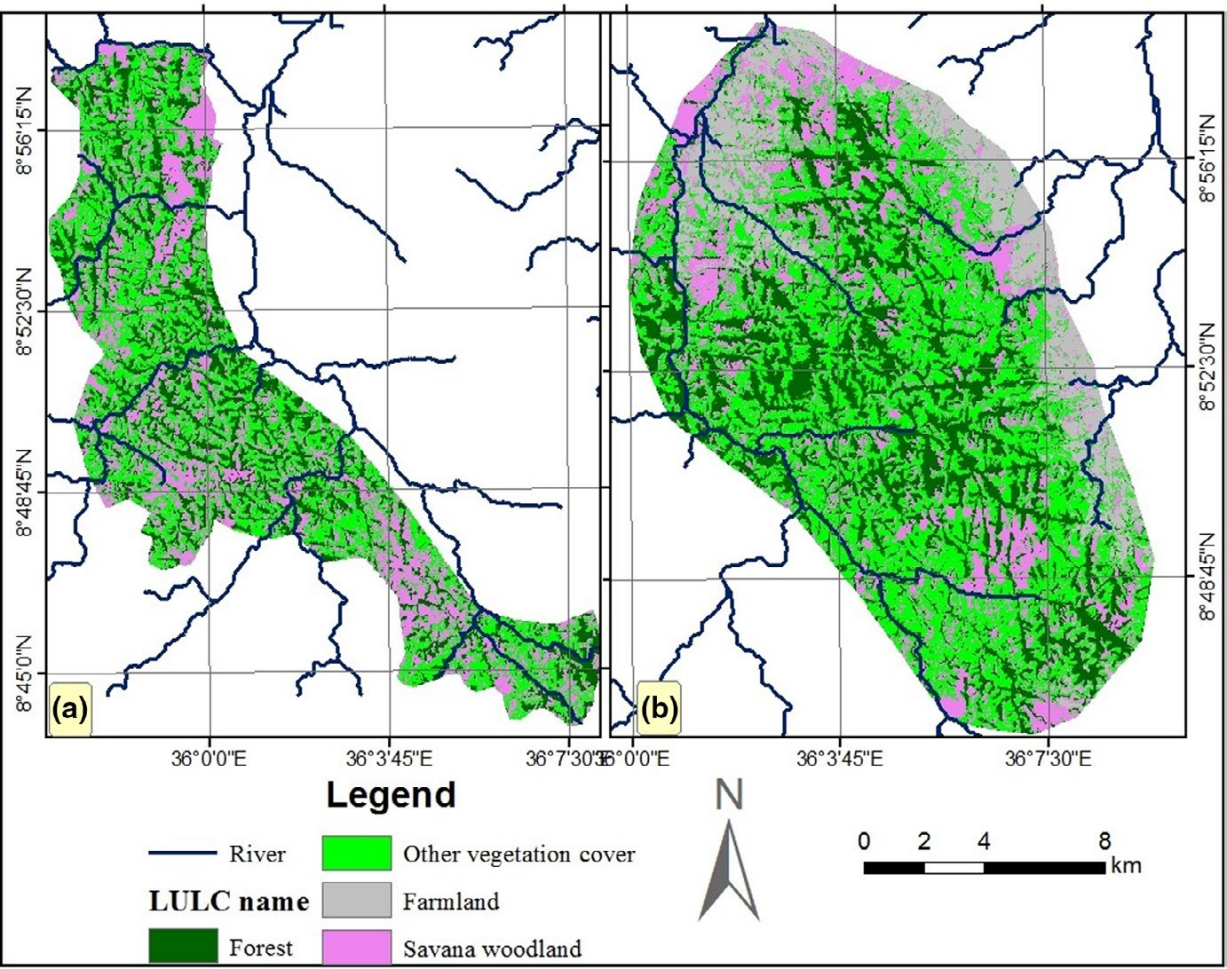
Land cover types of the PPWH (a) and HADCHA (b)

Land use land cover types of HADCHA and PPWH were classified based on the dominant land cover types of the area (Figure 8). The total area of HADCHA is greater than the total area of the PPWH around HADCHA. Besides, the size of each land cover type in HADCHA is larger than the respective land cover types in the PPWH. Though HADCHA and PPWH have similar land cover types, large amount of land (2,309.40 ha) in HADCHA was used for farmland, while only 4.20 ha was used in PPWH (Table 6).

**TABLE 6:** Land cover types of HADCHA and the PPWH in the 2019

LULC classes	HADCHA		PPWH	
	Area (ha)	Area (%)	Area (ha)	Area (%)
Savanna woodland	8,350.70	33.05	5,118.2	30.46
Forest	5,442.40	21.54	4,787.7	28.50
Farmland	2,309.40	9.14	4.2	0.02
Other vegetation covers	9,166.20	36.27	6,890.4	41.01
Total	25,268.70	100.00	16,800.13	100.00

## 4 DISCUSSION

### 4.1 Sighting locations of African buffalo

As described by Funston et al. (1994), seasonal variation in the availability of food and water alters the range and feeding habits of African buffalo. African buffalo spent much time around the riverine and riparian banks during the dry season due to the availability of forages. During the wet season, African buffalo were recorded at longer distances from HADCHA, and around Dabena river valley and Qoddi Gassi. These areas had been free from human disturbances but recently settlements are underway around Qoddi Gassi where African buffalo herds were commonly observed. The higher occurrence of African buffalo in PPWH suggested that buffalo potentially use the area as it has comparable habitat requirements for large mammals such as lion, warthog, bush pig, hippopotamus, bushbuck, waterbuck, and many more nonhuman primates. During the periods of trophy hunting in HADCHA, most large mammals including African buffalo move out of HADCHA, and less accessible for trophy hunters as they take refuge in the PPWH. This could make trophy hunters unsuccessful because they are not allowed to move out of the range for trophy hunting. On the contrary, this has created a good opportunity for poachers and local trophy hunters to easily access bushmeat species and African buffalo in PPWH. The expansion of HADCHA toward the current PPWH could have paramount significance to conserve other excluded wildlife habitats and resources thereby reduce illegal trophy hunting of African buffalo practiced in PPWH. Moreover, African buffalo is one of the big ungulates that require a large home range for foraging and other life activities. This could have been the cause for the movement of African buffalo out of HADCHA.

### 4.2 Habitats features used in wildlife habitat mapping

Adjacent to HADCHA, large potential wildlife habitats were left unprotected. This made most large mammals vulnerable to bushmeat and illegal trophy hunters in the unprotected habitats. In this study, wildlife habitats were considered to be suitable if they have good cover or shelter, food, slope <35°, and have good access to water as reported by Bailey et al. (1996). They stated that abiotic factors such as slope, distance to water, distance to shade or thermal cover, temperature, wind, and other barriers can influence foraging patterns thereby habitat preferences of mammals. For instance, livestock prefers to graze gentle terrain than a steeper slope. Most herbivores refrain from grazing in areas with slopes greater than 20% because it needs more energy for up and down locomotion (Gillen et al., 1984). Besides, steep slope areas less likely to hold moisture to produce fresh and palatable forage during the dry season. However, habitats closer to the river and water sources are highly preferred by mammals compared with habitats far from water sources. This could allow many wild mammals to easily access water sources without traveling a longer distance. As revealed by Gillen et al. (1985), riparian habitats attracted herbivores due to large amounts of nutritious, palatable forage, moderate slope gradient, reliable water supply, and more favorable microclimate. Traveling longer distances in search of food and water may expose herbivore prey to predators, and costly in terms of energy and time. Wildlife habitats closer to human‐induced pressure such as road, built‐ups, agricultural activities, fallow lands, and other disturbed lands in and around wildlife habitats were categorized as unsuitable because human activities could disturb the normal activities of animals in the wild. As reported by Zarri et al. (2008), slope, terrain, elevation, and distance to settlements seem to have a significant impact on habitat suitability and habitat selection of large mammals. The road is an important factor for habitat degradation and reduction in habitat suitability for animals (Wilkie et al., 2000).

### 4.3 Habitat suitability of HADCHA versus PPWH

This study revealed highly suitable wildlife habitats in both HADCHA and PPWH. Out of the total areas analyzed, about 77% of HADCHA was identified as suitable and 9.75% as highly suitable. This could be a confirmation for the good status of HADCHA though about 9.14% of its total area was seriously disturbed by a human, and totally unsuitable. The unsuitability of the habitat is due to the expansion of agricultural activities through shifting cultivation. Agriculture and settlement are the important parameters of disturbance that lead to habitat fragmentation thereby change the suitability status of wildlife habitats (Lindenmayer & Fischer, 2006). Moreover, a significant amount of HADCHA (4%) was still categorized as moderately suitable. This could be ascribed to the various anthropogenic activities carried out in the forest such as livestock grazing and illegal resource extractions. About 16,800 ha PPWH was identified and mapped as suitable for medium and large‐sized mammals. These areas were relatively free from human built‐ups and agricultural activities compared with HADCHA. However, about 0.03% and 2.84% of the areas were categorized as unsuitable and moderately suitable, respectively. Though the habitat suitability of HADCHA and the PPWH are not the same, both have comparable habitat suitability which could support medium‐ and large‐sized mammals. Large mammals require a large home range for grazing and other activities. If the size of a given protected area is small, large mammals move to the edge or out of the protected area and are the causes of human–wildlife conflicts. Hence, expanding the ranges of large mammals through the investigation of potential wildlife habitat could minimize human–wildlife conflicts around protected areas.

### 4.4 Land use/land cover types analysis

The HADCHA is typically characterized by lowland vegetation except for the upper escarpments which encompass shrubland, wooded savannah, and broad‐leaved vegetation types. Because of an increased human population around the midland and highland areas, and large free space in the lowland areas, people migrate to the lowland areas for subsistence agriculture and livestock rearing. As stated by Vreugdenhil et al. (2012), people migrate toward the borders of protected areas seeking fertile soil and livestock grazing land in Ethiopia. This could have a significant negative impact on wildlife habitats around protected areas. Wildlife habitats modified by human settlements and farmlands are irreplaceable and have long‐lasting negative impacts on wildlife and their habitats (Mc Granahan & Satterthwaite, 2003).

In HADCHA savanna woodland, the dominant vegetation cover decreased, whereas forest, farmland, and other vegetation covers increased between the years 2009 and 2019. For instance, the savanna woodland decreased by 2,934.61 ha (35.14%) between the years 2009 and 2019. On the contrary, farmlands, human settlements, grazing land, and fallow land increased by 885.39 ha (38.34%) between the years 2009 and 2019. This revealed that it is the savanna woodland habitat that is highly modified into farmland, human settlements, grazing land, and other vegetation covers. The modification of savanna woodland for the purpose of human need has a serious impact on large herbivore mammals because it serves as a good grazing land for herbivores than forested habitats. Due to loosening of law enforcement, most protected areas in Ethiopia are poorly managed and thus many protected grasslands are used for livestock grazing, subsistence farming, and commercial agriculture (Young, 2012).

### 4.5 Land cover types of HADCHA and PPWH

The total area of HADCHA is greater than the areas of newly PPWH. However, the PPWH comparably has more habitat suitability than HADCHA because the new wildlife habitat was marked relatively free of human‐dominated landscapes. For instance, in the newly PPWH, 4.2 ha (0.02%) were identified as unsuitable (farmland, settlements, grazing, and fallow land). However, in HADCHA, 2,309.40 ha (9.14%) of land was identified as unsuitable because the edges were converted into farmland through shifting cultivation.

## 5 CONCLUSION

Geospatial technologies are used to gather information about the physical parameters of wildlife habitats and geospatial modeling for wildlife habitat evaluation. Such technologies are preferred because they are economical in terms of time and cost to provide accurate data in the conservation and management of wildlife and their habitats. This study attempted to address the applications of GIS to identify and map potential wildlife habitats around HADCHA. African buffalo were considered as species of concern to map the potential wildlife habitats because it is a species for which the area was established as a controlled hunting area. The result suggested that PPWH that could be used for the conservation of wildlife was identified and mapped in the western parts of HADCHA. The PPWH could be used as an ideal place for the conservation and management of medium‐ and large‐sized mammals. The PPWH has more suitable habitats than HADCHA though its overall area is less than HADCHA. HADCHA was under severe human pressure such as shifting cultivation and livestock grazing. Hence, the identified and mapped potential wildlife habitats around HADCHA shall be legally protected and added into HADCHA so as to increase the home range of African buffalo and other potential trophy species in the area. Moreover, the legal protection of neglected areas adjust to HADCHA could be helpful for the sustainable conservation and use of wildlife and their habitats in the future.

## CONFLICT OF INTEREST

None declared.

## AUTHOR CONTRIBUTIONS

**Mosissa Geleta Erena:** Conceptualization (equal); Data curation (equal); Formal analysis (equal); Funding acquisition (equal); Investigation (equal); Methodology (equal); Project administration (equal); Resources (equal); Software (equal); Supervision (equal); Validation (equal); Visualization (equal); Writing‐original draft (equal); Writing‐review & editing (equal). **Tekalegn G. Yesus:** Conceptualization (equal); Data curation (equal); Formal analysis (equal); Funding acquisition (equal); Investigation (equal); Methodology (equal); Project administration (equal); Resources (equal); Software (equal); Supervision (equal); Validation (equal); Visualization (equal); Writing‐original draft (equal); Writing‐review & editing (equal).

## Data Availability

Data about the accuracy assessment of LULC classification and habitat suitability maps of this study are available from the Dryad Digital Repository (https://doi.org/10.5061/dryad.w3r2280r4).
